# Emergence of CXCR4-tropic HIV-1 variants followed by rapid disease progression in hemophiliac slow progressors

**DOI:** 10.1371/journal.pone.0177033

**Published:** 2017-05-04

**Authors:** Tsunefusa Hayashida, Kiyoto Tsuchiya, Yoshimi Kikuchi, Shinichi Oka, Hiroyuki Gatanaga

**Affiliations:** 1 AIDS Clinical Center, National Center for Global Health and Medicine, Tokyo, Japan; 2 Center for AIDS Research, Kumamoto University, Kumamoto, Japan; "INSERM", FRANCE

## Abstract

**Objective:**

The association between emergence of CXCR4-tropic HIV-1 variants (X4 variants) and disease progression of HIV-1 infection has been reported. However, it is not known whether the emergence of X4 variants is the cause or result of HIV-1 disease progression. We tried to answer this question.

**Design:**

HIV-1 *env* sequences around the V3 region were analyzed in serially stocked samples in order to determine whether X4 variants emerged before or after the fall in CD4+ T-cell count.

**Methods:**

The study subjects were five HIV-1-infected hemophiliac slow progressors. Deep sequencing around the HIV-1 *env* V3 region was conducted in duplicate. Tropism was predicted by geno2pheno [coreceptor] 2.5 with cutoff value of false positive ratio at <5%. When X4 variant was identified in the latest stocked sample before the introduction of antiretroviral therapy, we checked viral genotype in previously stocked samples to determine the time of emergence of X4 variants.

**Results:**

Emergence of X4 variants was noted in two of the five patients when their CD4+ T-cell counts were still high. The rate of decrease of CD4+ T-cell count or of rise of HIV-1 load accelerated significantly after the emergence of X4 variants in these two cases. Phylogenetic analysis showed that these X4 variants emerged from CCR5-tropic HIV-1 viruses with several amino acid changes in the V3 region.

**Conclusions:**

The emergence of X4 variants preceded HIV-1 disease progression in two hemophiliac slow progressors.

## Introduction

Tropism of HIV-1 is defined by the usage of coreceptor on human cells, and has been considered to be associated with disease progression [1]. Many studies have reported that primary HIV-1-infected patients mainly have CCR5-tropic HIV-1 viruses (R5 viruses) while about half of the patients in the late stage of HIV-1 infection have both R5 viruses and CXCR4-tropic HIV-1 variants (X4 variants) [2]. In addition, the association between emergence of X4 variants and rapid reduction of CD4+ T-cell counts has been reported [3–8]. The X4 variants seem to be exposed to stronger immune pressure and tend to be more sensitive to neutralizing antibodies compared with R5 viruses [9–11]. Previous reports demonstrated the disappearance of the X4 variants following the development of primary HIV-1 infection with a mixture of R5 viruses and X4 variants [12,13]. Thus, the existence of X4 variants is considered unlikely under sufficient host immunity. However, X4 variants can reemerge after deterioration of host immunity. Evidence suggests that X4 variants have a more potent ability to destroy the immune system because they have higher replication capacity in some cells and a wider range of host cells than R5 viruses [1,14,15]. Although the natural course of tropism change has been well studied [7,16–18], it is not clear yet whether the emergence of X4 variants is a cause or result of falling CD4+ T-cell count [2].

HIV-1 has reverse transcriptase with low fidelity, which allows a variety of not only interindividual but also intraindividual viral RNA sequences [19]. Especially, intraindividual variety of viral sequence generates quacispecies, which contribute to the development of drug resistance, escape from host immunity, and change in tropism [20–22]. Previously, sub-cloning of PCR products was the only technique to analyze the quacispecies, though there was a limitation of the number of clones that could be analyzed. Recently, the development of deep sequencing has provided the chance to identify minor populations of quacispecies [23–29]. In addition, genotype assays have been developed to predict viral tropism, and the results of these assays are in agreement with the results of phenotype assays, which are time-consuming and costly [8,30–36]. In this study, we examined five HIV-1 slow progressors to determine whether the emergence of X4 variants is the cause or result of disease progression.

## Materials and methods

### Subjects

We focused on hemophiliac HIV-1-infected patients to find slow progressors of HIV-1 infection. In this study, we defined patients who were antiretroviral therapy (ART)-naïve over 20 years with HIV-1 infection as slow progressors. In Japan, 1432 hemophiliacs acquired HIV-1 infection through contaminated blood products in the early 1980s [37]. By 2015, 310 HIV-1 infected hemophiliac patients had visited the AIDS Clinical Center, National Center for Global Health and Medicine, Japan. Among them, 80 patients regularly visited the center in 2015. Among the latter group, 5 patients were HIV-1 slow progressors, and all were included in this study after providing signed informed consent. The ethics committee of National Center for Global Health and Medicine approved the collection and analysis of the samples. We had previously analyzed tropism in one (Case 1) of these 5 patients and the results were published previously [38].

### Deep sequencing

HIV-1 RNA was extracted from plasma or serum samples stored at -80°C using High Pure Viral RNA Kit (Roche Diagnostics, Mannheim, Germany) or High Pure Viral Nucleic Acid Large Volume Kit (Roche Diagnostics). Human DNA was extracted from peripheral blood mononuclear cells (PBMC) stored at -80°C using QIAamp DNA Mini Kit (Qiagen, Tokyo, Japan). Viral sequences around the V3 region (position in HXB2: 6970–7314) of HIV-1 *env* were analyzed after amplification by nested PCR. RT-PCR was conducted using PrimeScript High Fidelity RT-PCR Kit (Takara Bio, Shiga, Japan). Subsequently, a second PCR was conducted using PrimeStar Max DNA Polymerase (Takara Bio). The primers of the first PCR were forward 106A (5’ CAT ACA TTA TTG TGC CCC GGC TGG 3’) and reverse C3E (5’ AGA AAA ATT CCC CTC TAC AAT TAA 3’). The primers of the second PCR were forward C2 (5’ (adapter and others)—AAT GTC AGC ACA GTA CAA TGT ACA C 3’) and reverse 10B (5’ (adapter and others)—ATT TCT GGG TCC CCT CCT GAG G 3’). The primers of the second PCR had adapter, key, and multiple identifiers at the 5’ end for the purpose of emulsion PCR and subsequent pyrosequencing, based on the information provided by the manufacturer (http://www.454.com/). The final PCR products were purified by Agencourt AMPure XP (Beckman Coulter, Brea, CA). The sample was quantified using 2100 Bioanalyzer (Agilent Technologies, Santa Clara, CA) with High Sensitivity DNA Kit (Agilent Technologies), and by 7900HT (Thermo Fisher Scientific, Kanagawa, Japan) with KAPA Library Quantification Kits (Kapa Biosystems, Wilmington, MA). Deep sequencing was conducted with GS Junior (Roche Diagnostics).

### Data analysis

Data of deep sequencing were analyzed by Amplicon Valiant Analyzer (Roche Diagnostics) with cutoff values of ≥2 reads and ≥0.2%. Then, homopolymeric errors were collected manually. To evaluate manual collection of homopolymeric errors, we calculated the error rate in our method using monoclonal viral RNA that was prepared by transfection of plasmid pNL4-3, which possesses full length HIV-1 genome [39]. Briefly, Opti-MEM I (Thermo Fisher Scientific) and Lipofectamine 2000 (Thermo Fisher Scientific) were used to transfect pNL4-3 into 293T cells. The supernatant was collected after 3 days, and viral RNA was extracted from the supernatant [40]. Viral tropisms were predicted by geno2pheno [coreceptor] 2.5 with cutoff value of false positive ratio (FPR) at <5% [41]. First, we checked viral tropism in the latest and ART naïve samples from each patient. When X4 variants were identified in the sample, we then checked viral sequences with the previously stocked samples to determine the time of emergence of X4 variants. Sequences were aligned by clustalW, and phylogenetic analysis was conducted by MEGA 7.0.14 using the neighbor-joining method and the Kimura 2-parameter model with 100 bootstrap replications. The diversity of nucleic acids around the V3 region was calculated in each analyzed sample by weighted average pairwise difference using MEGA 7.0.14. Linear regression analysis was performed using GraphPad Prism version 4.00 for Windows (GraphPad Software, La Jolla, CA). T-test and interaction test of multiple regression analysis, which was used for comparison of the slope, were performed using IBM SPSS Statistics software version 23 (IBM Japan, Tokyo).

## Results

### Error rate of deep sequencing

First, we checked the error rate of our deep sequencing method using monoclonal viral RNA prepared by transfection of pNL4-3 [39]. The results of Amplicon Variant Analyzer showed 26,547 reads of 345 bp sequences and 34,422 bases of error (S1 Table). Thus, the error rate was 3.76 × 10^−3^. Among these errors, 34,238 errors (99.5%) were 1-base long or short homopolymeric errors caused by continuing A. Then, we collected these homopolymeric errors manually. After that, 184 errors remained and the final error rate was 2.01 × 10^−5^.

### Identification of X4 variants

**Fig 1:**
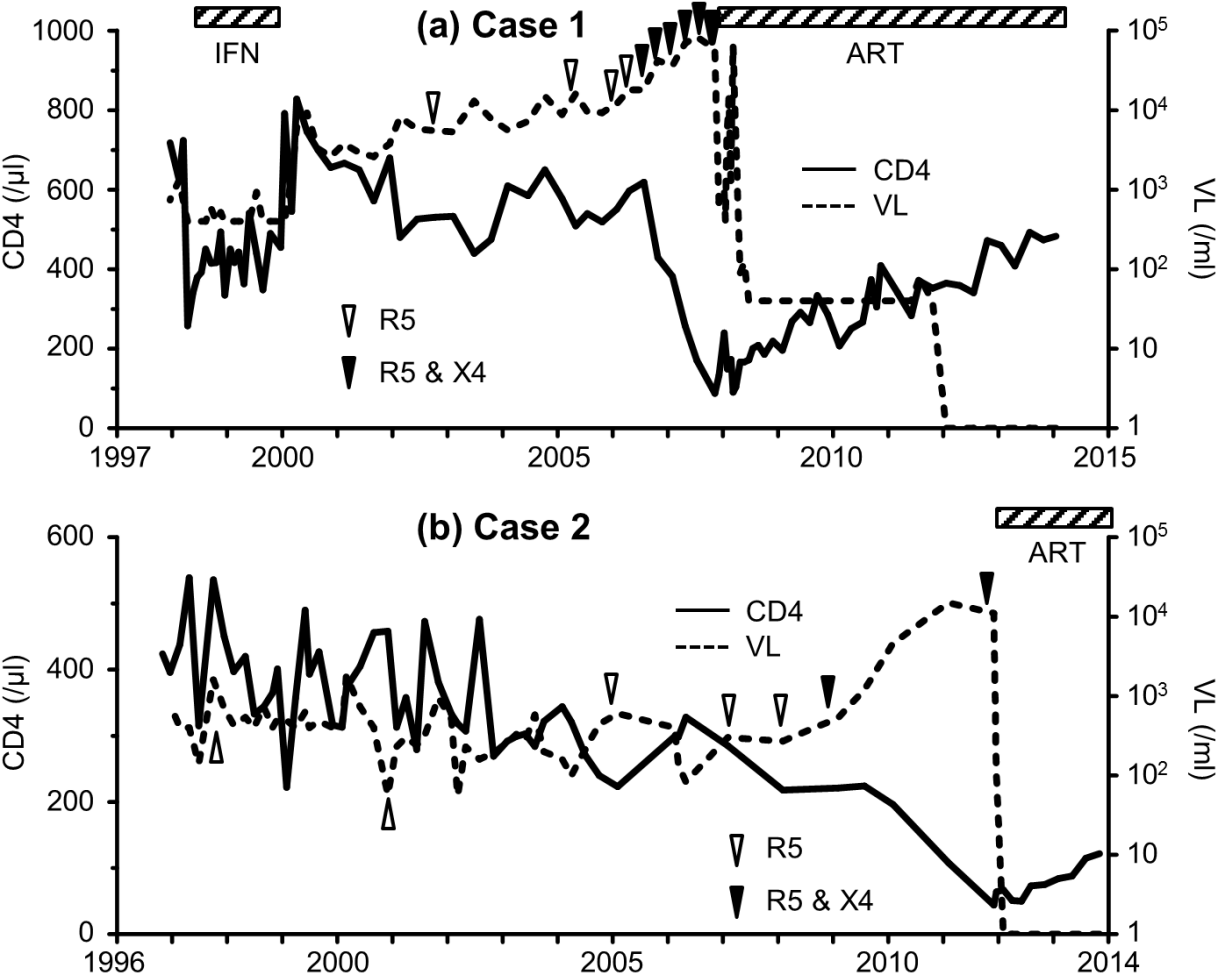
Clinical course of two hemophiliac HIV-1 slow progressors with emergence of X4 variants. Changes in CD4+ T-cell count and HIV-1 viral load are shown for Case 1 (a) and 2 (b). Triangles represent the results of tropism predicted by deep sequencing. Interferon therapy was provided for treatment of chronic hepatitis C between April 1998 and October 1999. Antiretroviral therapy was initiated in November 2007 and November 2011 in Case 1 and 2, respectively. ART: anti-retroviral therapy, INF: interferon, VL: viral load.

We checked the HIV-1 sequence in the latest samples obtained from the five hemophiliac slow progressors stocked before the introduction of ART (Fig 1 and S1 Fig). In Case 1, the PBMC sample collected in November 2007 was used to check the viral sequence. At that time, the CD4+ T-cell count was 88/μL and HIV-1 viral load (VL) was 58000/mL. Deep sequencing was performed twice, with a mean read number of 10816 and 17.4% of them were identified as X4 variants. In Case 2, the serum sample collected in November 2011 was used for the analysis (CD4+ T-cell count was 44/μL and VL was 11000/mL). The mean read number was 4813 and 16.0% of them were identified as X4 variants. In Case 3, we analyzed a serum sample collected in January 2013 (CD4+ T-cell count was 289/μL and VL was 27000/mL). The mean read number was 2531 and only R5 viruses were identified. In Case 4, a serum sample collected in August 2011 was used (CD4+ T-cell count was 264/μL and VL was 10000/mL). The mean read number was 3376 and only R5 viruses were identified. In Case 5, a serum sample collected in August 2012 was used (CD4+ T-cell count was 363/μL and VL was 6900/mL). The mean read number was 1848 and all were R5 viruses.

The two patients with X4 variants (Cases 1 and 2) had significantly lower CD4+ T-cell count (mean 66/μL) compared with the other three patients without X4 virus (mean 305/μL, *p* = 0.011, by t-test). On the other hand, the VL was not different (*p* = 0.550) between the two patients with X4 variant (mean 34,500/mL) and the three patients without X4 variants (mean 14,633/mL). In the two patients with X4 variants, we also checked the viral sequences using previously stocked samples to determine the time of emergence of the X4 variants (Fig 1A and 1B and Table 1). The first emergence of X4 variants was July 2006 (CD4+ T-cell count was 619/μL) in Case 1 and January 2009 (CD4 count was 221/μL) in Case 2. Deep sequencing was performed in duplicate except for Case 2’s samples collected in January 2007 and January 2008. We were able to amplify HIV-1 sequences only one time in these samples, probably due to the low VL (300 and 270/mL, respectively, Table 1).

**Table 1:** Samples and results of deep sequencing in Cases 1 and 2.

	Date	Type of samples	CD4 (/μl)	VL (/ml)	Mean read number	Mean % of X4 virus
Case 1	Oct 2003	serum	475	7800	25327	0
	Apr 2005	PBMC	509	16000	2735	0
	Jan 2006	plasma	551	12000	5786	0
	Apr 2006	PBMC	597	18000	36757	0
	Jul 2006	plasma	619	18000	17582	0.9
	Oct 2006	PBMC	429	42000	6661	3.8
	Jan 2007	PBMC	382	36000	6290	23.3
		serum			6533	4.3
	Apr 2007	serum	257	69000	2644	8.6
	Jul 2007	serum	171	84000	8751	0.7
	Nov 2007	PBMC	88	58000	10816	17.4
Case 2	Sep 1997	serum	536	1600	4011	0
	Oct 2001	serum	313	230	2516	0
	Jan 2005	serum	223	600	3476	0
	Jan 2007	serum	285	300	1385*	0*
	Jan 2008	serum	218	270	3231*	0*
	Jan 2009	plasma	221	530	3118	90.5
	Nov 2011	serum	44	11000	4813	16.0

* PCR was successful only once, thus these results represent one deep sequencing only.

VL: viral load.

The dataset of the deep-sequencing was deposited in DNA databank of Japan with the accession number LC225774-LC227557.

### Speed of CD4 reduction and VL elevation

**Fig 2:**
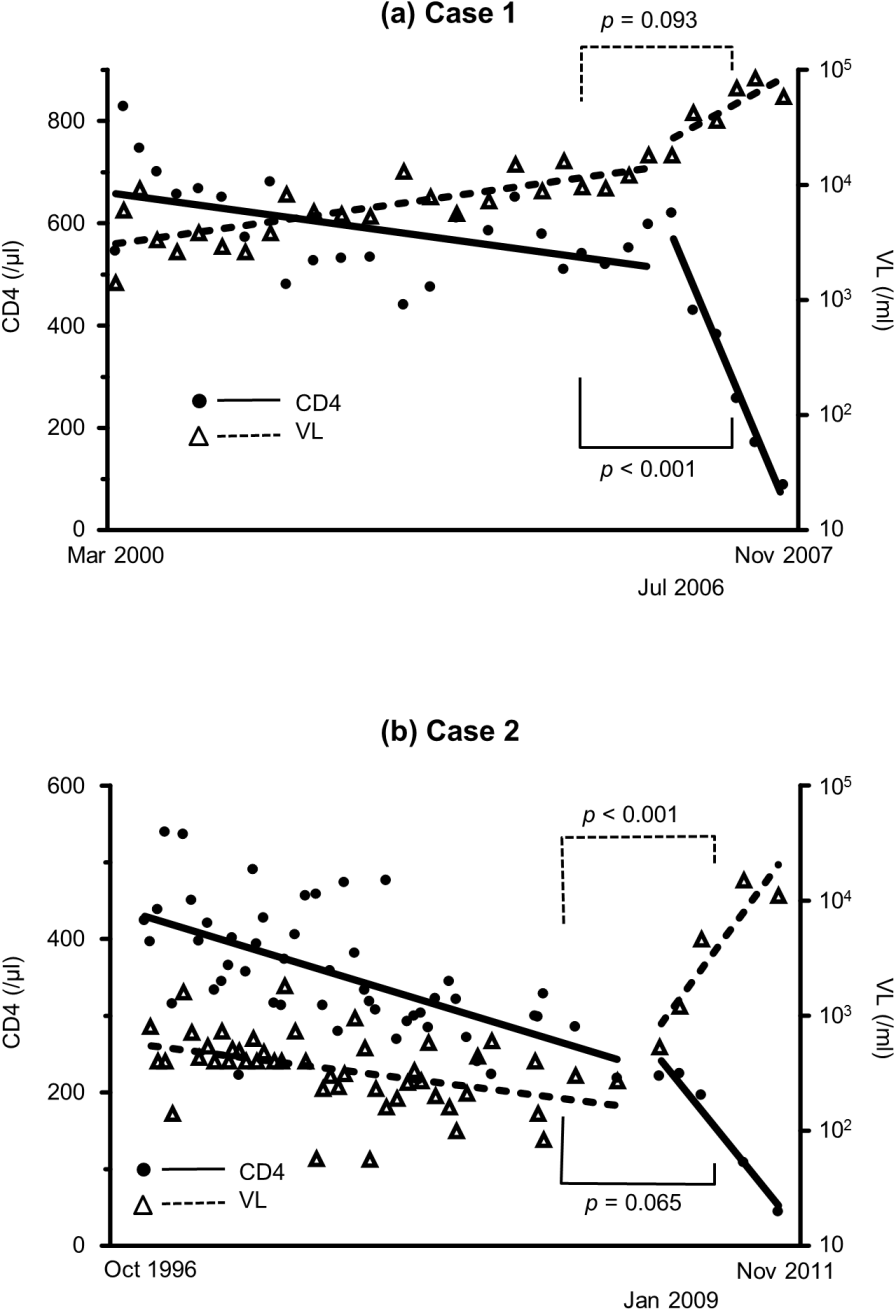
Slopes of CD4+ T-cell count and VL in two patients who showed emergence of X4 variants. Data and slopes of linear regression analysis of CD4+ T-cell count and HIV-1 viral load during the observation period in Case 1 (a) and 2 (b). Statistical analysis was performed with interaction test of multiple regression analysis between the two slopes before and after the emergence of X4 variants. VL: viral load.

**Fig 3:**
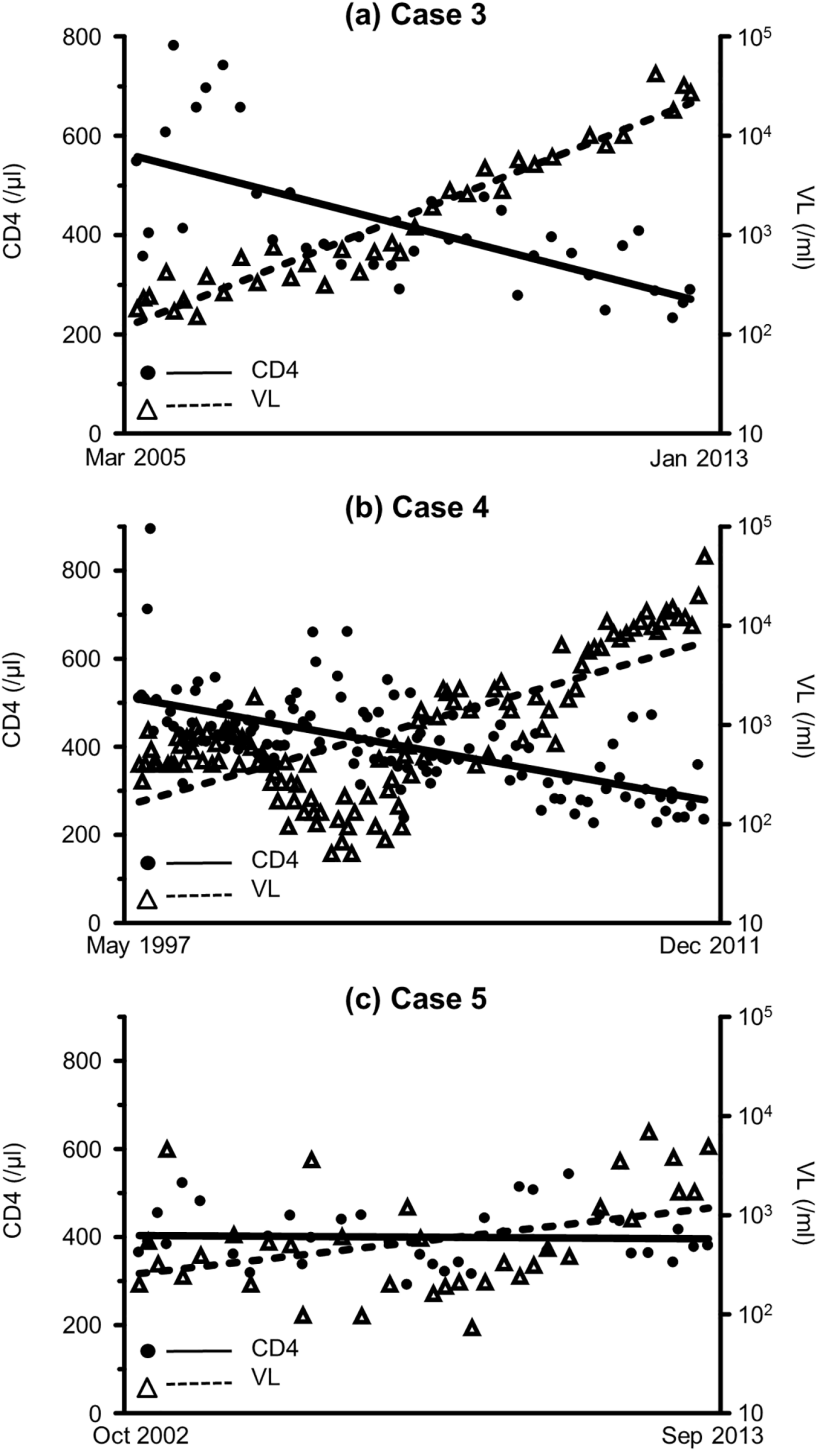
Changes in the slope of CD4+ T-cell count and that of VL in the three patients who did not show emergence of X4 variants. Data and slopes of linear regression analysis of CD4+ T-cell count and HIV-1 viral load during the observation period of Case 3 (a), 4 (b), and 5 (c). Statistical analysis was performed with interaction test of multiple regression analysis between the two slopes before and after the emergence of X4 variants. VL: viral load.

To investigate whether the emergence of X4 variants affected disease progression, we checked CD4+ T-cell count and VL before and after the emergence of X4 variants. The data within the duration of interferon treatment for HCV or of ART for HIV-1 were excluded from this analysis because interferon usually reduces both CD4+ T-cell count and VL [42]. In Case 1, the slope of the linear regression of CD4+ T-cell count was -23.2/μL/year before April 2006, and -403/μL/year after July 2006 (*p*<0.001, by interaction test of multiple regression analysis, Fig 2A). Furthermore, the slope of the linear regression of log VL was 0.107/mL/year before April 2006, and 0.413/mL/year after July 2006 (*p* = 0.093). In Case 2, the slope of the linear regression of CD4+ T-cell count was -16.6/μL/year before January 2008 and -67.6/μL/year after January 2009 (*p* = 0.065, Fig 2B), while the slope of the linear regression of log VL was -0.0466/mL/year before January 2008, and 0.495/mL/year after January 2009 (*p*<0.001). In comparison to Case 1, the CD4 count was already low (221/μL) at the emergence of X4 variants in Case 2, which may explain why the difference in the slope of CD4 decrease did not reach significant level. Furthermore, after the emergence of X4 variants, the rate of VL increase changed significantly only in Case 2 but not in Case 1, which may be explained by the already high VL (18000/ml) in Case 1. In both cases, the disease progression rate increased after the emergence of X4 variants. In Cases 3, 4, and 5, who had no X4 variants, the slopes of the linear regression of CD4+ T-cell count were -36.4, -15.7, and -0.684/μL/year, respectively (Fig 3A–3C), whereas the slopes of the linear regression of log VL were 0.280, 0.111, and 0.0601/mL/year, respectively.

### Phylogenetic analysis

**Fig 4:**
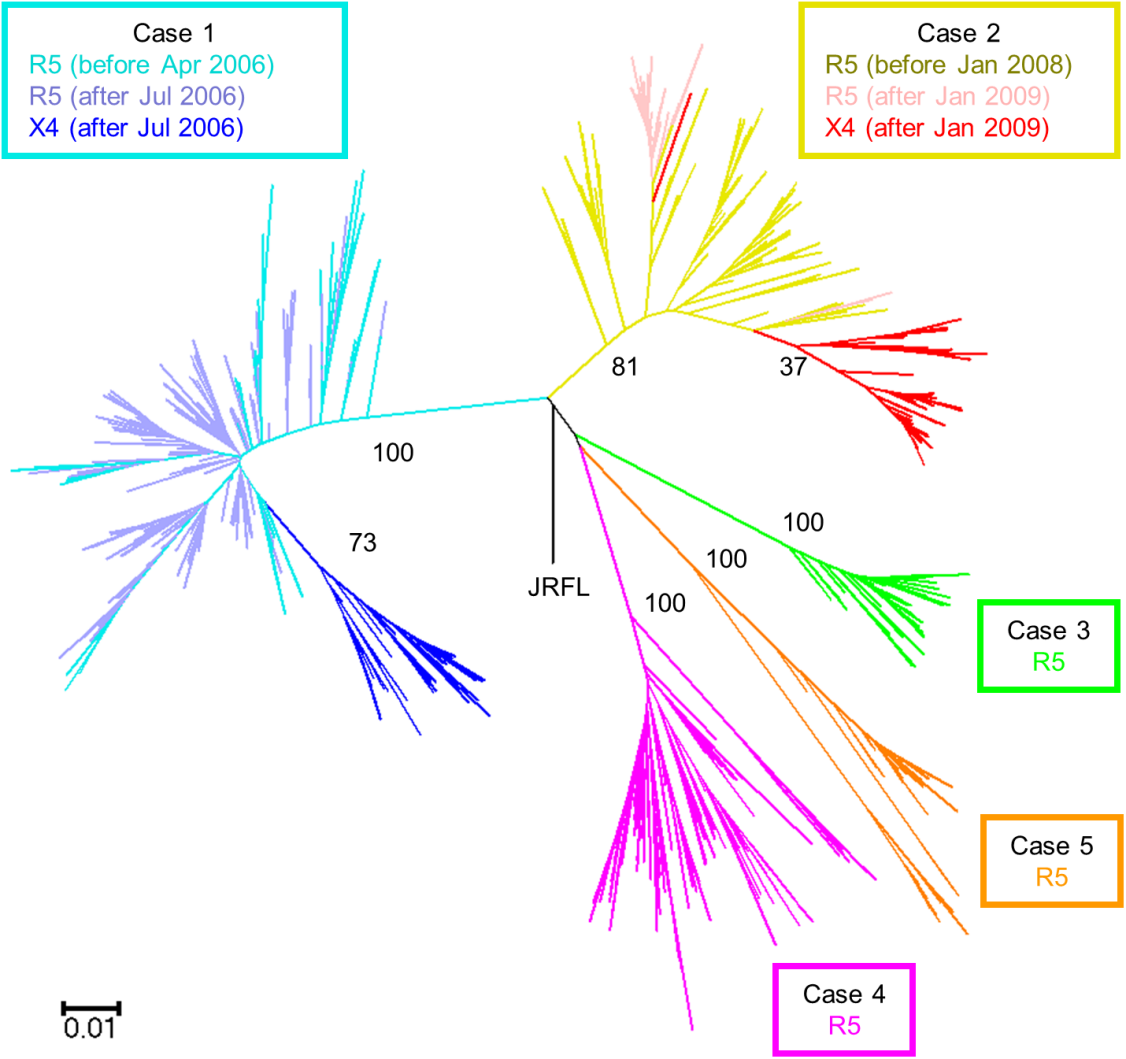
Phylogenetic analysis of deep sequencing using 345 bp (position in HXB2: 6970–7314) around the V3 region of HIV-1 env in five hemophiliac HIV-1 slow progressors. Phylogenetic tree was constructed using the neighbor-joining method and the Kimura 2-parameter model with HIV-1 JRFL sequence as an out-group. The R5 sequences before and after the emergence of X4 variants are shown for Case 1 in light-blue and purple, respectively, while the X4 sequences are shown in dark-blue. R5 sequences before and after the emergence of X4 variants are shown for Case 2 in yellow and pink, respectively, while the X4 sequences are shown in red. The sequences of Cases 3, 4, and 5 are shown in green, orange, and magenta, respectively. Numbers represent bootstrap values.

To determine the origin of X4 variants, phylogenetic analysis was performed with the results of deep sequencing. A total of 315,743 valid sequences were obtained from the five patients with GS Junior. The amplicon variant analyzer merged identical sequences, resulting in 1784 different sequences, which were used in phylogenetic analysis. All the sequences were identified as HIV-1 subtype B. Using 345 bp around the V3 region, phylogenetic analysis showed significant cluster in each patient, and aggregation of X4 variants in Cases 1 and 2 (Fig 4). Several characteristic mutations in the V3 region were evident in the X4 variants of Cases 1 and 2 (S2 and S3 Figs). In Case 1, X4 variants first emerged in July 2006, and they showed four common amino acid changes [T8L, K10I, S11RH (insertion), and D29N], which had never been seen before. Subsequently, T8V appeared in January 2007, then T8L was completely displaced by T8V in April 2007. In Case 2, X4 variants first emerged in January 2009 and the majority had 5 amino acid changes (K10E, S11R, P13S, T22A, and E25R). The K10E, S11R, and E25R changes have never been seen before. Phylogenetic analysis indicated that the X4 variants evolved from R5 viruses by acquiring several amino acid changes in the V3 region and that once X4 variants emerged, they began to evolve rapidly resulting in their specific clusters, while the R5 virus continued to evolve independently from X4 variants in the phylogenetic tree. The calculated diversity around the V3 region continued to increase and the trend of the increase did not change significantly at the emergence of X4 variants in both Case 1 and 2 (S2 Table).

## Discussion

Although many studies have reported the association between emergence of X4 variants and rapid progression of HIV-1 disease, it is still not clear whether the emergence of X4 variants is the cause or result of disease progression of HIV-1 infection [2]. In order to answer this question, it is necessary to follow the natural course of untreated HIV-1-infected patients before ART until the emergence of X4 variants. Inevitably, observation under substantially long-term ART-free period is necessary. However, current treatment guidelines strongly recommend early introduction of ART following the diagnosis of HIV-1 infection [43], and it is usually difficult to postpone the commencement of ART until the emergence of X4 variants. In this study, we included five treatment-naïve slow progressors who acquired HIV-1 infection in early 1980s [37] and visited our outpatient clinic regularly. We successfully identified the emergence of X4 variants and analyzed the sequential changes in the *env* V3 region in two of these patients. In both cases, a rapid increase in HIV-1 viral load associated with CD4 decline was observed after the emergence of X4 variants, necessitating the start of ART. Thus, it seems that the emergence of X4 variants is not the result but the cause of HIV-1 disease progression.

The X4 variants emerged from R5 viruses with several amino acid changes in the V3 region. How did these characteristic mutations occur? It is possible that these mutations occurred simultaneously though they occurred more likely at different times. They may rather have accumulated one by one, though the intermediate V3 sequences (those with only one or two mutations) were not identified in the present study. Considering the detection limit of our assay (0.2%), the mutations may have accumulated in extremely small number of viruses. Actually, the sequence of one X4 variant was separated from the cluster of the other X4 sequences in Case 2 in the phylogenetic tree (Fig 4). This does not suggest that the evolution from R5 to X4-tropic variants occurred twice in this patient, because the X4 sequence harbored the same characteristic mutations (K10E, S11R, P13S, T22A, and E25R in V3 region) as the other X4 variants. It is more reasonable to consider that the mutations accumulated in viruses with minor population below the detection limit or at sites other than blood cells, such as the lymph nodes. It is probable that the intermediate viruses during the evolution had low replication ability [44]. It is conceivable that the virus with complete set of the characteristic mutations carried competitive replication ability by acquiring compensatory mutations. The likely scenario includes the emergence of X4 variants with full replication ability, which was followed by a fall in the host CD4+ cell count.

It is difficult to determine the causal relationship between the emergence of X4 variants and HIV-1 disease progression although such emergence was followed by rapid CD4+ T-cell decline and VL increase in our study. Since our findings were limited to only two cases and they were hemophiliac slow progressors, one cannot generalize these findings to all patients with HIV-1 infection. Another limitation of our study is that we confirmed the tropism phenotypically only in the viruses of Case 1 in our previous study [38] but not in the viruses of Case 2. However, the analyzed sequences here were typical subtype B and the identified X4 variants in both Cases 1 and 2 harbored a substitution of a basic amino acid at the 11th position of the V3 region (S11R), which is common in X4 variants following the 11/25 rule [35,45,46]. Therefore, it is reasonable to conclude that they were actually X4 tropic viruses.

Admittedly, the X4 variants did not always emerge in every patient before the CD4+ cell decline. However, after the emergence of X4 variants, CD4+ cell count started to decrease rapidly even in slow progressors. Further studies on the pathogenesis of X4 variants are necessary in order to clarify the effects of viral tropism on clinical outcome, though careful long-term observation of the natural course of HIV-1 disease is ethically difficult.

## Supporting information

S1 Fig Clinical course of three hemophiliac HIV-1 slow progressors who did not show emergence of X4 variants. ART: anti-retroviral treatment. INF: interferon (anti-HCV treatment). VL: viral load.
(TIF)

S2 Fig Amino acid sequences of the V3 region in Case 1. Percentage values represent the percentage of the sequence in each sample. FPR: false positive ratio.
(TIF)

S3 Fig Amino acid sequences of the V3 region in Case 2. Percentage values represent the percentage of the sequence in each sample. FPR: false positive ratio.
(TIF)

S1 Table Errors of deep sequencing with monoclonal HIV-1 RNA prepared from pNL transfection. Position shows the location of errors in the 345 bp fragment of PCR amplicon. Total reads were 26,547.
(DOCX)

S2 Table Diversity around the V3 region in Cases 1 and 2. Data show the calculated nucleic acid diversity.
(DOCX)
