# Supplementary figures and images for: Emergence of CXCR4-tropic HIV-1 variants followed by rapid disease progression in hemophiliac slow progressors
Source: PLoS One. 2017 May 4;12(5):e0177033. doi: 10.1371/journal.pone.0177033 (PMC5417636; doi:10.1371/journal.pone.0177033)

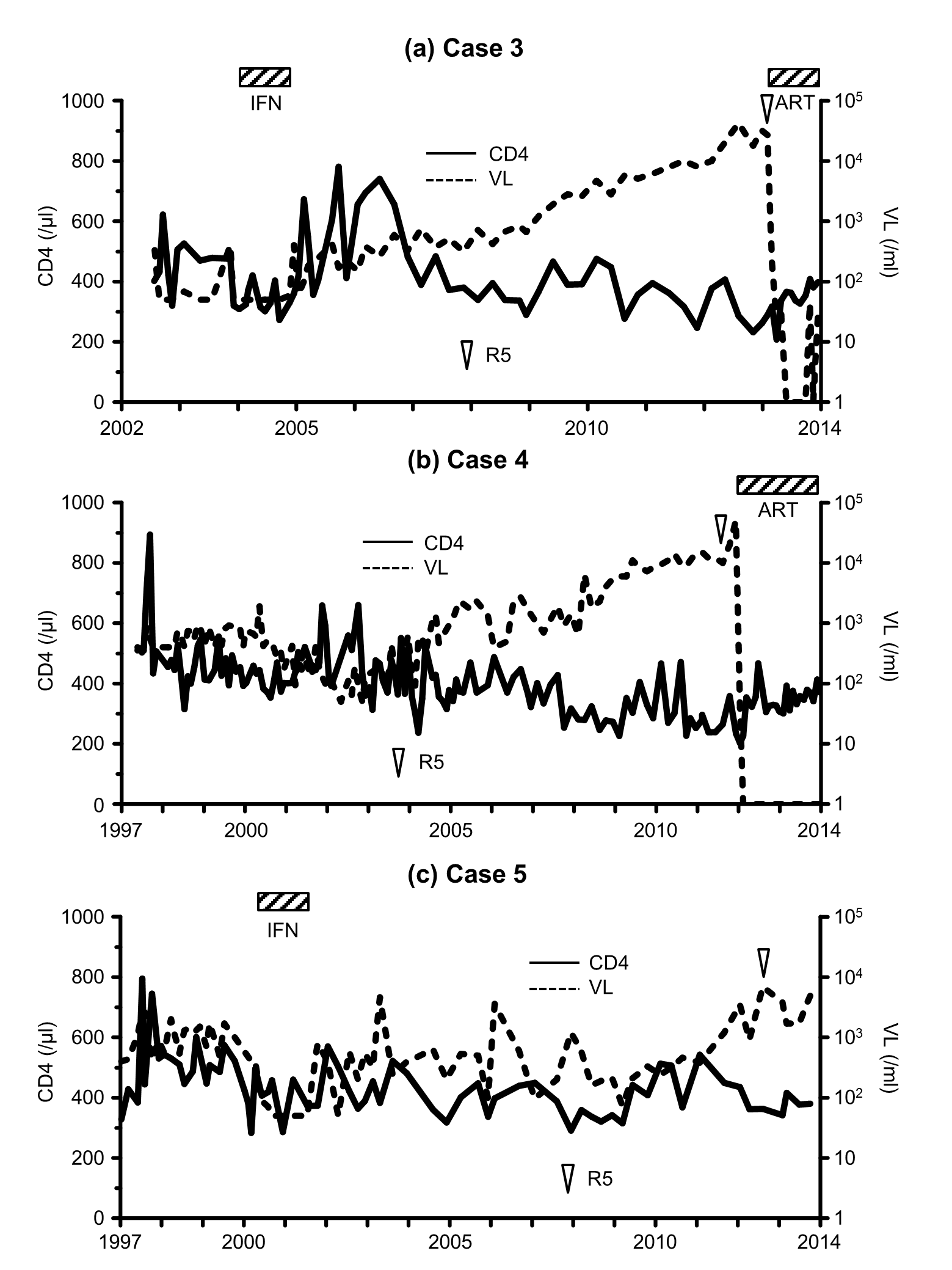

Supplement: S1 Fig — ART: anti-retroviral treatment. INF: interferon (anti-HCV treatment). VL: viral load. (TIF) [file pone.0177033.s001.tif]

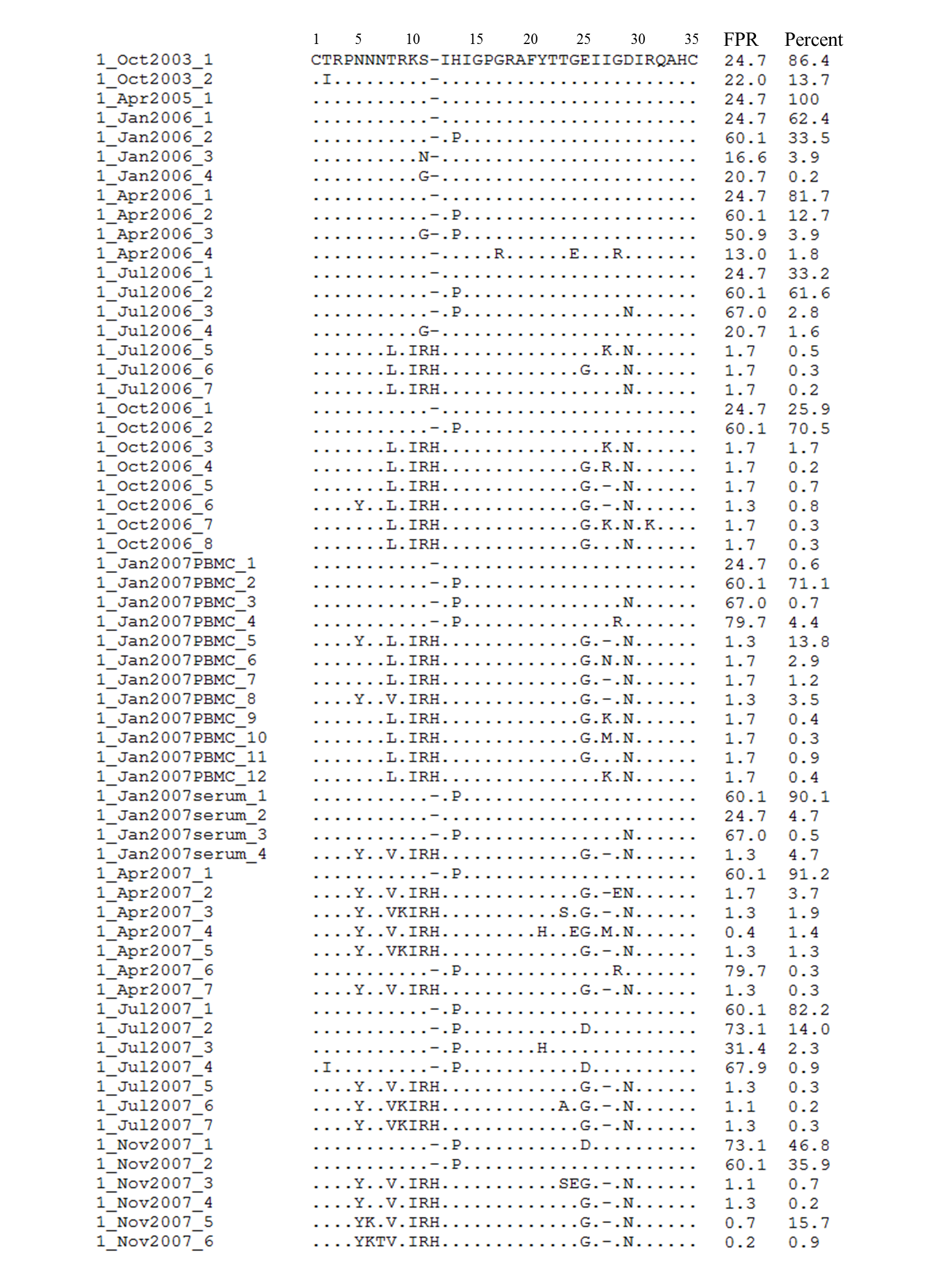

Supplement: S2 Fig — Percentage values represent the percentage of the sequence in each sample. FPR: false positive ratio. (TIF) [file pone.0177033.s002.tif]

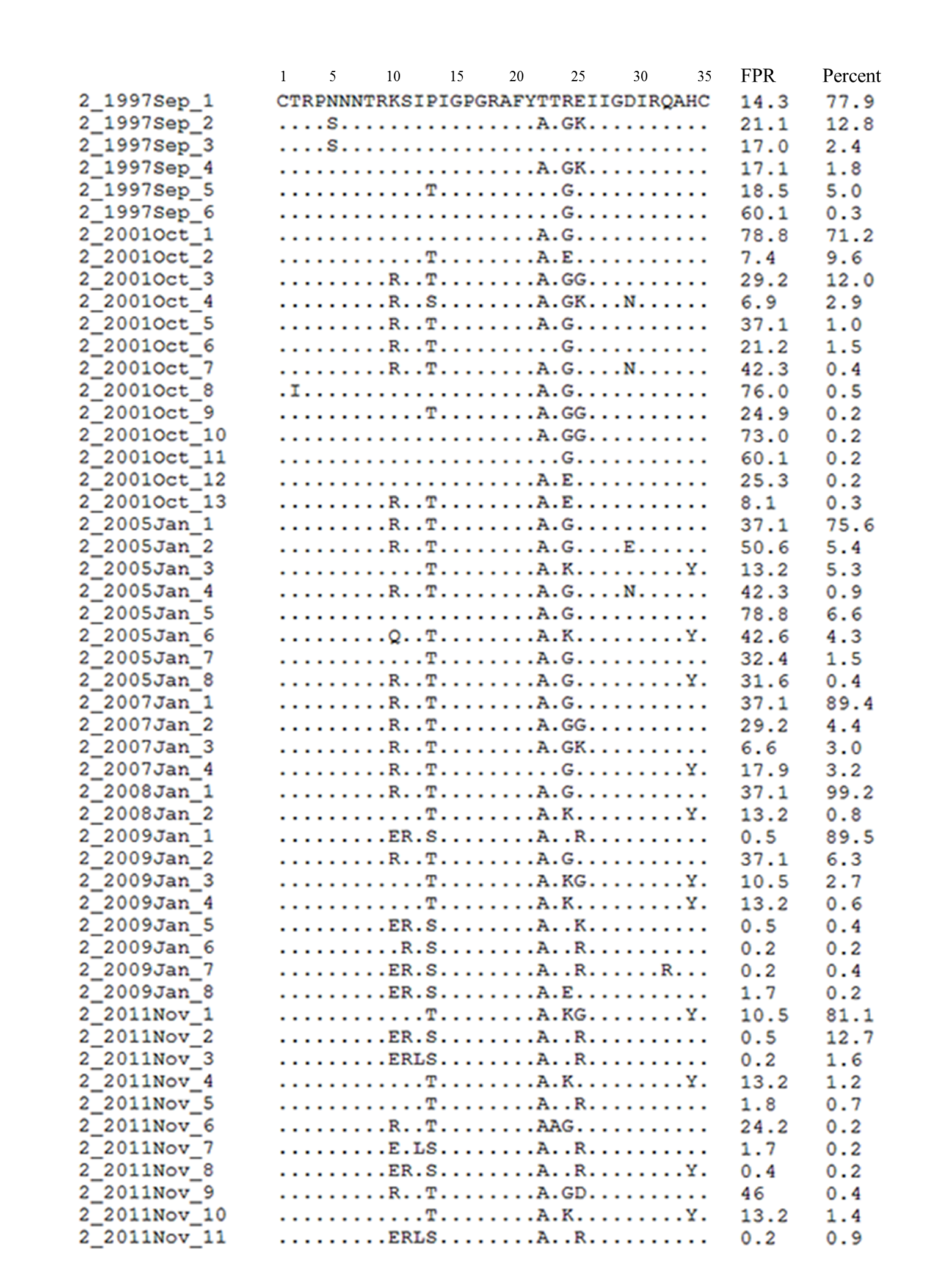

Supplement: S3 Fig — Percentage values represent the percentage of the sequence in each sample. FPR: false positive ratio. (TIF) [file pone.0177033.s003.tif]
