# Supplementary material for: Emergence of CXCR4-tropic HIV-1 variants followed by rapid disease progression in hemophiliac slow progressors
Source: PLoS One. 2017 May 4;12(5):e0177033. doi: 10.1371/journal.pone.0177033 (PMC5417636; doi:10.1371/journal.pone.0177033)
Supplement: S1 Table — Position shows the location of errors in the 345 bp fragment of PCR amplicon. Total reads were 26,547. (DOCX) [file pone.0177033.s004.docx]

**S1 Table. Errors of deep sequencing with monoclonal HIV-1 RNA prepared from pNL transfection.**

| Position | Sequence | number of reads | Type | Error rate (%) |
| --- | --- | --- | --- | --- |
| 171 b | AAAAAG | 967 |  |  |
|  | AAAAG | 25580 | short | 96.4 |
| 222 b | AAAAAT | 25422 |  |  |
|  | AAAAT | 537 | short | 2.0 |
|  | AAAAAAT | 472 | long | 1.8 |
|  | AAAAACT | 116 | insertion | 0.4 |
| 311 b | AAT | 25903 |  |  |
|  | AAAT | 644 | long | 2.4 |
| 319 b | AAAT | 26428 |  |  |
|  | AAAAT | 119 | long | 0.4 |
| 322 b | AAT | 25207 |  |  |
|  | AT | 1340 | short | 5.0 |
| 327 b | AAAAC | 25973 |  |  |
|  | AAAAAC | 574 | long | 2.2 |
| 330 b | AAT | 25796 |  |  |
|  | AAAT | 644 | long | 2.4 |
|  | AT | 107 | short | 0.4 |
| 333 b | AAT | 23892 |  |  |
|  | AAAT | 2565 | long | 9.7 |
| 340 b | AAG | 25397 |  |  |
|  | AAAG | 1150 | long | 4.3 |
| 342 b | GC | 26479 |  |  |
|  | GTC | 68 | insertion | 0.3 |
| 344 b | AAT | 26041 |  |  |
|  | AAAT | 506 | long | 1.9 |

Position shows the location of errors in the 345 bp fragment of PCR amplicon. Total reads were 26,547.
