# Supplementary material for: Emergence of CXCR4-tropic HIV-1 variants followed by rapid disease progression in hemophiliac slow progressors
Source: PLoS One. 2017 May 4;12(5):e0177033. doi: 10.1371/journal.pone.0177033 (PMC5417636; doi:10.1371/journal.pone.0177033)
Supplement: S2 Table — Data show the calculated nucleic acid diversity. (DOCX) [file pone.0177033.s005.docx]

**S2 Table. Diversity around the V3 region in Cases 1 and 2.**

| Case | Date | Type of samples | Mean diversity | Tropism |
| --- | --- | --- | --- | --- |
| 1 | Oct 2003 | serum | 0.0261 | R5 |
|  | Apr 2005 | PBMC | 0.0001 | R5 |
|  | Jan 2006 | plasma | 0.0315 | R5 |
|  | Apr 2006 | PBMC | 0.0231 | R5 |
|  | Jul 2006 | plasma | 0.0295 | R5 & X4 |
|  | Oct 2006 | PBMC | 0.0309 | R5 & X4 |
|  | Jan 2007 | PBMC | 0.0347 | R5 & X4 |
|  |  | serum | 0.0242 | R5 & X4 |
|  | Apr 2007 | serum | 0.0235 | R5 & X4 |
|  | Jul 2007 | serum | 0.0230 | R5 & X4 |
|  | Nov 2007 | PBMC | 0.0283 | R5 & X4 |
| 2 | Sep 1997 | serum | 0.0188 | R5 |
|  | Oct 2001 | serum | 0.0161 | R5 |
|  | Jan 2005 | serum | 0.0223 | R5 |
|  | Jan 2007 | serum | 0.0191* | R5 |
|  | Jan 2008 | serum | 0.0157* | R5 |
|  | Jan 2009 | plasma | 0.0197 | R5 & X4 |
|  | Nov 2011 | serum | 0.0246 | R5 & X4 |

Diversity was calculated by weighted average pairwise difference using MEGA 7.0.14.

* PCR was successful only once, thus these results represent a single deep sequencing only.
